# Contributions of Nonleaf Organs to the Yield of Cotton Grown with Different Water Supply

**DOI:** 10.1155/2014/602747

**Published:** 2014-06-01

**Authors:** Dongxia Zhan, Ying Yang, Yuanyuan Hu, Yali Zhang, Honghai Luo, Wangfeng Zhang

**Affiliations:** The Key Laboratory of Oasis Ecology Agriculture of Xinjiang Production and Construction Group, Shihezi University, Shihezi 832003, China

## Abstract

The objectives of this experiment were (i) to determine the effect of water supply on the photosynthetic capacity of leaves, bracts, capsule walls, and stalks of cotton at different growth stages and (ii) to determine the contributions of these nonleaf organs to whole plant photosynthesis. Water deficit reduced the total surface area per plant but increased the proportion of nonleaf to total plant surface area. Net photosynthetic rates of leaves declined rapidly beginning 25 days after anthesis. In contrast, the net photosynthetic rates of bracts and capsule walls were insensitive to soil moisture stress and decreased by a small amount between 25 and 45 days after anthesis. The relative contribution of bracts and stalks to canopy apparent photosynthesis (CAP) increased under water deficit conditions. Cotton seed weight in the conventional irrigation treatment decreased by 10.1–29.7% when the bolls (capsule walls plus bracts) were darkened and by 5.3–9.9% when the stalks were darkened. On a percentage basis, both boll photosynthesis and stalk photosynthesis contributed more to seed weight when the plants were grown under water deficit conditions rather than nondeficit conditions. In conclusion, nonleaf organs contribute significantly to yield when cotton plants are under water stress during late growth stages.

## 1. Introduction

Most studies about photosynthesis have focused on the contribution of leaves. However, other plant parts can retain or develop chlorophyll. Evidence of photosynthetic activity has been found in petioles, stems [1], wood and bark [2], and roots [3]. Reproductive organs, such as flowers, seeds [4, 5], and developing fruit [6, 7], are also photosynthetically active.

In cotton (*Gossypium hirsutum* L.), bracts, capsule walls, and stems are capable of photosynthetic CO_2_ fixation. Photosynthesis in these plant parts can make a significant contribution to yield [8–11]. Morris [12] reported that when the capsule walls were shaded, with or without the removal of the bracts, the weight of lint in the boll was reduced by about 30%. Removal of the bracts without shading the boll wall caused no reduction in lint weight. In contrast, Bhatt [13] observed that bract photosynthesis not only contributed to boll development but also regulated the transport of assimilate from the leaves. Recent observations by Du et al. [14] and Zhang et al. [15] indicated that both the main stems and the capsule walls of cotton are photosynthetically active and can contribute to canopy photosynthesis and yield. Some researchers have suggested that cotton leaves are limited in their ability to support fruit growth, particularly during periods of peak reproductive development [11, 16–18]. Hence, it is essential for the cotton plant to increase other possible sources of assimilate for fruit growth.

Several researchers have analyzed photosynthesis in nonleaf parts of cotton either from morphological, physiological, or C assimilation viewpoints [19–21]. However, there are no reports about the relative contribution of leaves, bracts, stalks, and capsule walls to canopy apparent photosynthesis (CAP) in cotton during fruit development, particularly with respect to the contribution of these nonleaf organs to seed development. An important research challenge is to maximize the photosynthesis potential of nonleaf organs under water deficit conditions. Therefore, the objectives of this study were (i) to determine the effect of water supply on the photosynthetic capacity of leaves, bracts, capsule walls, and stalks of cotton at different growth stages and (ii) to determine the contribution of nonleaf organs to whole plant photosynthesis.

## 2. Materials and Methods

### 2.1. Plant Materials and Growing Conditions

This experiment was conducted during the 2012 and 2013 growing seasons at an experimental field near Shihezi University, Xinjiang, China (45°19′ N, 86°03′ E) (Table 1). Three cotton cultivars (cv. Xinluzao 46, Xinluzao 45, and Xinluzao 33) were sown on April 18, 2012. We observed that the morphological and photosynthetic characteristics of Xinluzao 46 and Xinluzao 45 were fairly similar, so only two cotton cultivars (cv. Xinluzao 45 and Xinluzao 33) were sown on April 22, 2013. The row spacing was 12 cm and the plant density was 1.8 × 10^5^ plants hm^−2^. The plots were covered with plastic film mulch and drip irrigation was applied beneath the mulch. The plot size was 73 m^2^. Pest and weed control were carried out according to local practices.

The experiment consisted of three irrigation treatments: (i) conventional irrigation (CK), with the soil moisture content in the 0–60 cm depth maintained at 70–80% of field capacity throughout the growing season; (ii) slight deficit irrigation (T1), where the amount of irrigation water was 60–70% that in the CK treatment and the soil moisture content was approximately 50% of field capacity; and (iii) moderate deficit irrigation (T2), where the amount of irrigation water was 30–40% of that in the CK treatment and the soil moisture content was approximately 30% of field capacity. These treatments were similar to the division standards of soil water deficit described by Hsiao [22]. The experimental design was completely randomized with three replications. Soil moisture content in the 0–60 cm depth was monitored during the cotton growing season using watermark soil moisture sensors (model 200SS; Irrometer Co., Riverside, USA).

### 2.2. Experimental Methods

#### 2.2.1. Surface Area Measurement

The surface areas of leaves and bracts were measured using a leaf area meter (LI-3000C, Li-Cor, USA). The length and diameter of the stalks between the upper and lower leaves (including the carpopodium and the petiole) were measured using Vernier calipers. These measurements were used to calculate the surface areas of the stalks. The surface areas of the capsule walls were determined according to the method of Hu et al. [23]. We did not measure the surface areas of fallen leaves or other organs.

#### 2.2.2. Gas-Exchange Measurements

Gas-exchange parameters of the penultimate leaves, bracts, and capsule walls under the main leaf were determined with a portable photosynthesis instrument (Li-6400, Li-Cor, USA). A 2 cm × 3 cm chamber with a 6400-02B LED light source (1800 *μ*mol photons m^−2^ s^−1^) was used to measure the gas exchange of leaves and bracts. For whole fruits, we used a conifer chamber (6400-05, Li-Cor, USA) with a white LED light source (Luxeon LEDs; Electus Distribution, NSW, Australia). Four measurements were made for each plant organ. After the measurements were completed, the bracts and capsule walls were collected and their surface areas determined. The photosynthetic rates were then recalculated. The bolls were filled with seeds and fiber; therefore, the respiration rates of the bolls were higher than the net photosynthesis rates. For this reason, the net photosynthetic rates of the bolls were calculated by subtracting the respiration rate in the light from the respiration rate in the dark.

#### 2.2.3. Canopy Apparent Photosynthesis (CAP)

Canopy apparent photosynthesis was measured using the assimilation chamber method described by Reddy et al. [24] and Acock et al. [25]. The chamber was 90 cm long × 70 cm wide. The height of the chamber was adjusted depending on the height of the cotton plants. The acrylic film covering the chamber transmitted more than 95% of the sunlight. Two fans were installed inside the chamber to mix the air. Air temperatures within the chamber were monitored using automatic sensors. The measurements were made quickly after the chamber was set in place to prevent air leakage. Furthermore, two assistants held the chamber tightly against the soil surface.

The CO_2_ concentrations inside the chamber were measured with a LI-8100 soil CO_2_ flux system (Li-Cor, USA). The instrument was programmed to run automatically. We put the chamber in place over the plants and then monitored the CO_2_ concentrations inside the chamber. When the CO_2_ concentrations began to drop steadily, we began to record the measurements. The measurements were made for 60 s. The apparent photosynthetic rate of the whole canopy was measured first. Then, the chamber was removed and all of the leaves were excised from the plants. The chamber was replaced and the CO_2_ concentrations were measured as described above. The apparent photosynthetic rate of the leaves (i.e., canopy or ground surface area basis) was calculated by subtracting these results from the total canopy photosynthetic rate. The process was repeated step by step. The bracts were removed, then the bolls (without bracts), and finally the stalks. The CO_2_ concentrations inside the chamber were measured at each step. The apparent photosynthetic rate of each organ (on a canopy or ground surface area basis) was determined by subtraction. The measurements were replicated three times. We also measured soil respiration at each sampling point to correct the apparent photosynthetic rates. We excluded fallen leaves and other organs from our measurements.

#### 2.2.4. Determining the Contribution of Nonleaf Organs to Seed Weight

The relative contribution of nonleaf organs to seed weight was measured according to the method described by Araus et al. [26]. Briefly, the bolls (capsule walls plus bracts) of eight plants were covered with aluminum foil. We used a needle to make holes in the foil to allow for gas exchange. The holes were 1 mm-diameter at least 15 mm apart. The holes represented about 0.3% of the covered area. The foil covering was put in place 15 days after the bolls were formed and then left in place until harvest. Aluminum foil was also used to cover the stalks of eight other randomly selected plants. The stalks were covered from tip pruning until harvest. Eight plants with no covered parts were used as the control. The dry seed weight per boll was measured at harvest for boll-darkened, stalk-darkened, and control plants. In total, 72 cotton plants were used in this part of the experiment [(8 boll-darkened plants + 8 stalk-darkened plants + 8 control plants) × 3 water treatments]. The relative contribution of bolls and stalks to seed weight was calculated using the following equation:

(1) Relative  contribution(%)   =(control  yield−darkened  yield)control  yield×100.

### 2.3. Statistical Analyses

All data were subjected to analysis of variance (ANOVA) using SPSS 17.0 statistical software (SPSS Inc., Chicago, IL, USA). The significance of differences between mean values was determined with least significant difference (LSD) tests. Differences were considered significant at *P* < 0.05. The data are presented as the mean ± standard deviation.

## 3. Results 

### 3.1. Gas Exchange of Different Plant Parts

**Figure 1:**
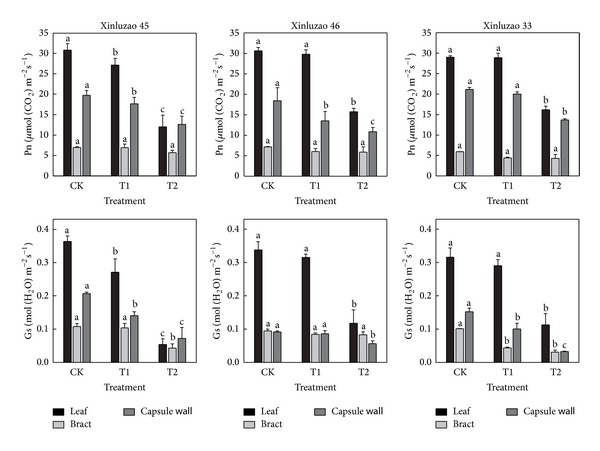
Gas-exchange parameters of leaves, bracts, and capsule walls in three cotton cultivars at the full boll stage in 2012. CK: conventional irrigation; T1: slight deficit irrigation; T2: moderate deficit irrigation. Different letters within a cultivar and within a plant organ are significantly different at the 0.05 probability level.

Net photosynthetic rates were higher in leaves than in either bracts or capsule walls at the full boll stage, regardless of soil water status (Figure 1). The T1 and the T2 treatments both reduced leaf photosynthesis and stomatal conductance. However, the negative effects of T2 were much larger than those of T1. Water deficit affected photosynthesis and stomatal conductance in leaves more than in bracts or capsule walls. Compared with the CK treatment, the T2 treatment reduced photosynthesis by 44.4–61.0% in leaves, 18.0–27.4% in bracts, and 35.3–41.0% in capsule walls. The patterns of stomatal conductance were similar to those of net photosynthesis in all treatments.

**Figure 2:**
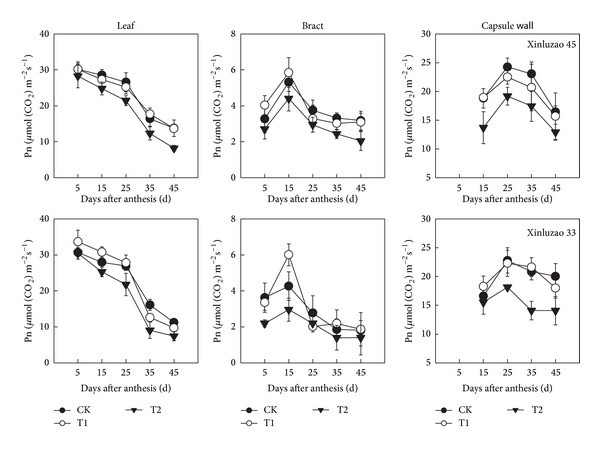
Temporal changes in the photosynthetic rates of leaves, bracts, and capsule walls in 2013. Values are means ± SD of four replicates. CK: conventional irrigation; T1: slight deficit irrigation; T2: moderate deficit irrigation.

On a surface area basis, the photosynthetic rates of leaves decreased significantly during boll development in 2013 (Figure 2). In comparison, the photosynthetic rate of bracts increased for the first 15 d after anthesis and then declined. The photosynthesis rates of capsule walls peaked 25 d after anthesis and then declined. The trends were the same for both cultivars. From the maximum to the minimum, photosynthetic rates declined most in leaves (54.3–75.5%) followed by capsule walls (40.2–68.9%) and then bracts (12.1–32.6%). During the late stage of boll development, the photosynthesis rates of capsule walls were higher than those of leaves. At 45 d after anthesis, the photosynthetic rates were between 7.4–8.2 *μ*mol m^−2^ s^−1^ for leaves, 1.4–2.0 *μ*mol m^−2^ s^−1^ for bracts and 12.9–14.1 *μ*mol m^−2^ s^−1^ for capsule walls. The photosynthesis rates of the main stem could not measure* in situ*.

Net photosynthetic rates of each plant organ at the full flower and the full boll stages are shown in Table 2. The photosynthetic rates of the three organs were added together within a growth stage to obtain the total photosynthetic rate. In the CK treatment, bracts and capsule walls accounted for 42.7–45.8% of the total photosynthetic rate at the full flower stage. At the full boll stage, bracts and capsule walls accounted for 58.5–61.6% of the total photosynthetic rate. The proportions in the T1 and T2 treatments were similar to those in the CK treatment.

### 3.2. Surface Areas of Leaf and Nonleaf Organs

The surface area of each plant organ was measured at the full flower and the full boll stages and then averaged together. The total surface area per plant (leaves + stalks + bracts + capsule walls) was significantly lower in both the T1 and T2 treatments than in CK treatment (Table 3). However, nonleaf organs (stalks + bracts + capsule walls) constituted a greater portion of the total surface area in the T1 and T2 treatments than in the CK treatment. On a percentage basis, stalks accounted for 15–20% of the total plant surface area, followed by bracts (11–18%) and then capsule walls (7–15%) (data not shown).

### 3.3. Canopy Apparent Photosynthesis in Plant Organs

On a ground surface basis, CAP of leaves and bracts decreased by a large amount during cotton fruit development. For example, between the early full boll and the medium boll open stage the CAP of leaves decreased by 80.6–88.7% in the CK treatment and the CAP of bracts decreased by 60.4–61.2%. In contrast, the CAP of stems decreased, but only by 13.1–25.6% during this period. The CAP values of bolls were negative, indicating that the canopy apparent respiration rates were high in bolls filled with seed and fiber. There was a positive association between the number of bolls per plant and the canopy apparent respiration rate (data not shown). The canopy apparent respiration values of bolls were higher in the CK treatment than in the T2.

Water deficit conditions resulted in a change in the relative contribution of leaves and nonleaf organs to CAP. At the full boll stage, leaves accounted for 65.3 to 67.0% of CAP in the T2 treatment compared with 74.6 to 78.5% of CAP in the CK treatment. In contrast, the relative contribution of nonleaf organs to CAP increased when the cotton plants were grown under water deficit conditions. For example, bracts accounted for 6.6 to 7.2% of CAP in the T2 treatment compared with 5.1 to 5.6% of CAP in the CK treatment. Stalks accounted for 5.3 to 7.8% of CAP in the T2 treatment compared with 4.1 to 4.9% of CAP in the CK treatment.

### 3.4. Relative Contribution of Nonleaf Organs to Yield

The relative photosynthetic contribution of bolls (capsule walls plus bracts) and the stalks to seed cotton dry weight per boll was assessed by darkening either the bolls or the stalks during fruit development (Table 4). Dry seed weight per boll in the CK treatment declined by 10.1–29.7% when the bolls were darkened and by 5.3–9.9% when the stalks were darkened. The seed weight declined even more when the bolls and stalks were darkened in the T1 and T2 treatments.

## 4. Discussion 

About 90–95% of crop biomass is derived from the products of photosynthesis. Leaves are considered to be the main photosynthetic organ; however, both bracts and capsule walls contain chlorophyll and are photosynthetically active [9, 10]. In the CK treatment of our study, the photosynthetic rates of bracts were 20.4–26.3% of those in leaves (on a surface area basis). The photosynthesis rates of capsule walls were 60.3–72.8% of those in leaves (Figure 1). Some differences would be expected depending on experiment conditions and varieties; however, overall, our results are similar to those of Wullschleger et al. [19] who observed that in cotton the photosynthetic rates of bracts and capsule walls were 10–40% of those in leaves.

In the CK treatment, the proportion of bracts and capsule walls net photosynthesis to the total increased significantly from the full flower stage to the full boll stage (Table 2). The trends were similar in all three water treatments. On a surface area basis, the photosynthetic rates of capsule walls were much higher than those of leaves at 25 d after anthesis (Figure 2). In a previous study, we observed that, *N* concentrations on a surface area basis were significantly higher in capsule walls than in leaves at about 20 days after anthesis [23]. Nitrogen is one of the main components of the photosynthetic apparatus [27]. Therefore, the relatively high photosynthetic rate of capsule walls during this period may be related to *N* concentration. Overall, these results indicate that photosynthesis by bracts and capsule walls has great importance to cotton yield, especially during the late growth stages.

**Figure 3:**
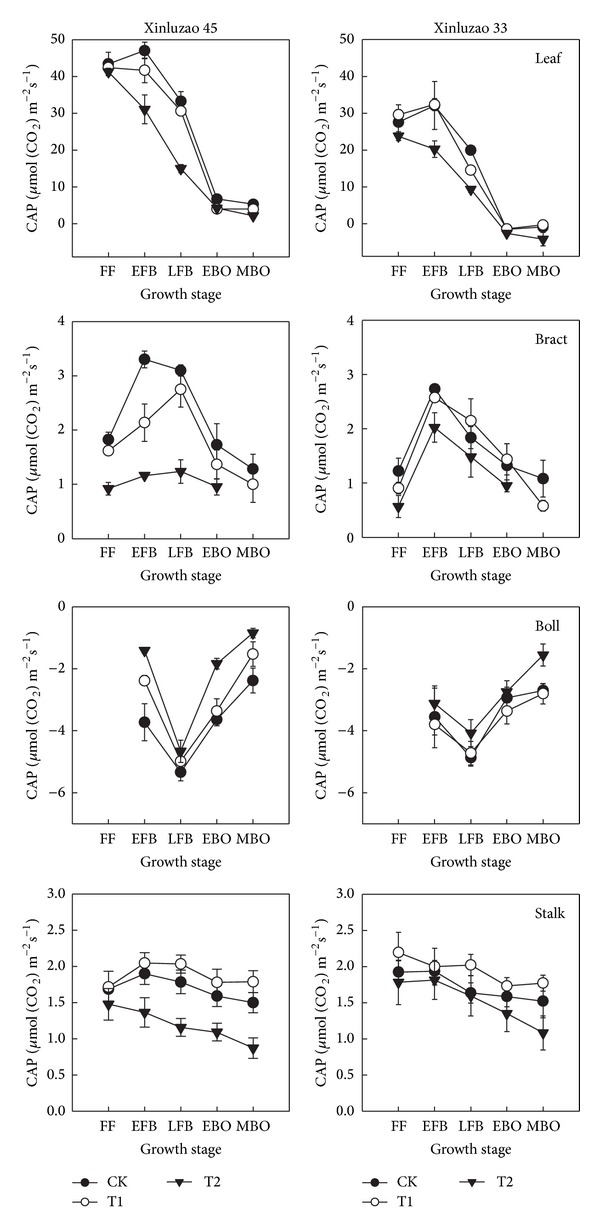
Temporal changes in canopy apparent photosynthesis of each cotton plant organ in 2013. CK: conventional irrigation; T1: slight deficit irrigation; T2: moderate deficit irrigation. FF: full flower; EFB: early full boll; LFB: late full boll; EBO: early boll open; MBO: medium boll open.

**Figure 4:**
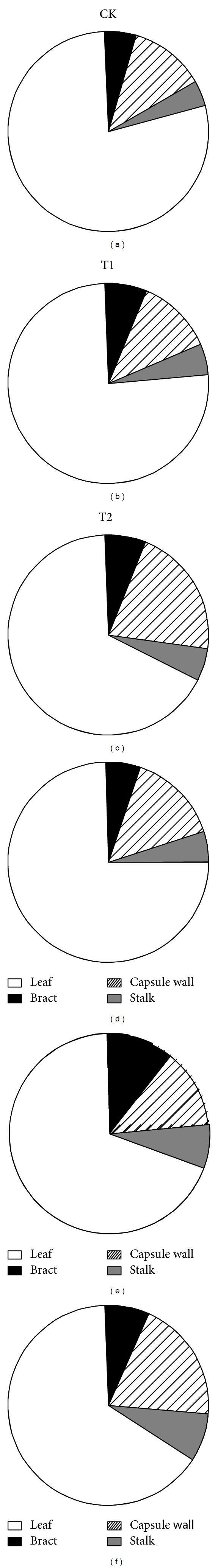
Relative contribution (%) of each plant organ to canopy apparent photosynthesis of Xinluzao 45 ((a)–(c)) and Xinluzao 33 ((d)–(f)) at the full boll stage. CK: conventional irrigation; T1: slight deficit irrigation; T2: moderate deficit irrigation. The contribution of fallen leaves or other plant parts was not taken into account.

Water deficit caused slight declines in the net photosynthetic rates of bracts and capsule walls (Figure 1). In contrast, the net photosynthetic rates of leaves decreased by a much larger amount. These results are similar to previous reports about the effect of water deficit on photosynthesis in winter wheat [28]. We observed similar trends in CAP. Specifically, after the early full boll stage, apparent photosynthetic rates decreased more quickly in leaves than in either bracts or stalks, regardless of irrigation amount (Figure 3). This indicated that nonleaf organs of cotton plants are insensitive to soil moisture stress. Depending on the water treatment, leaves accounted for 65.3–78.5% of CAP (Figure 4). Nonleaf organs accounted for the remaining 21.5–34.7% of CAP. This indicates that these nonleaf organs were a sizeable potential source of photosynthate. Capsule walls and bracts are near cotton fiber and seeds, which mean that photosynthate produced by these organs only needs to be transported a short distance [23]. Furthermore, Aschan and Pfanz [29] pointed out that nonleaf organs refixed 10%–85% of the CO_2_ produced by respiration. Our results suggest that leaf photosynthesis is supplemented by photosynthesis in stems, bracts, and capsule walls. This is especially important when leaf photosynthesis capacity declines due to aging or water stress.

Green surface area is an important index of crop photosynthesis. For example, the sepals of* Helleborus viridis* L. can account for 56% of the total plant surface area in the early spring [30]. Although floral organs constitute a relatively small fraction of the entire biomass, the green patterned inner tepals of* Galanthus nivalis* L. contribute significantly to photosynthetic activity [31]. Few studies have been done to measure the surface area of nonleaf organs in the cotton canopy. We observed that the proportion of nonleaf surface area to total surface area per plant increased as water deficit increased when averaged over both growing seasons (Table 3). These results suggest that increasing the surface area of nonleaf organs, which maintain their photosynthesis rates for longer periods than leaves and are insensitive to water stress, would compensate for the loss of leaf photosynthetic activity at the late full boll stage.

The relative contribution of different plant organs to yield can be estimated by darkening treatments [26, 32]. Darkening the bolls (capsule walls plus bracts) and stalks had significant effect on boll weight in our study. Specifically, seed weight in the CK treatment decreased by 10.1–29.7% when the bolls were darkened and by 5.3–9.9% when the stems were darkened (Table 4). Our findings are very similar to the results of Hu et al. (2012). The contribution of bolls and stalks to seed dry weight increased when cotton was grown under water-limiting conditions. It has been estimated that ear photosynthesis in cereals contributes 10–76% of final grain weight [32–34]. The exact contribution varies depending not only on genotype, but also on the experimental procedures and the environment.

## 5. Conclusions

Because of the early senescence of leaves, we suggest that it is important to increase the surface area of nonleaf organs. Both net photosynthetic capacity and canopy photosynthesis decreased less in bracts and capsule walls than in leaves. The contribution of boll and stalk photosynthesis to seed weight increased as water deficit increased. Leaves are the main organ contributing to yield formation; however, the contribution of nonleaf organ photosynthesis to cotton yield should not be ignored, especially during the late growth stages or when the plants are growing under stress conditions. We conclude that photosynthesis in nonleaf organs can make a significant contribution to cotton seed yield formation, especially when water stress occurs during fruit development.

## Figures and Tables

**Table 1:** Volumetric soil water content (%) at three depths and at three times during the 2012 and 2013 growing seasons.

Growth stage	Depth (cm)	2012			2013		
		CK	T1	T2	CK	T1	T2
Full flower	0–20	13.4	11.5	8.7	15.2	14.3	13.0
	20–40	11.9	9.6	8.0	15.1	13.6	13.0
	40–60	10.1	8.5	7.7	15.0	12.5	10.6

Full boll	0–20	14.6	13.0	11.1	17.3	16.5	13.6
	20–40	13.9	10.4	9.8	17.2	15.0	13.1
	40–60	10.2	8.1	6.5	16.1	13.1	11.9

Boll open	0–20	12.5	10.6	9.1	16.3	15.8	12.2
	20–40	11.6	8.6	7.9	15.7	13.4	10.3
	40–60	9.4	7.9	7.1	16.6	12.9	9.9

CK: conventional irrigation; T1: slight deficit irrigation; T2: moderate deficit irrigation.

**Table 2:** Photosynthetic rates of whole leaves, whole bracts, and whole capsule walls at the full flower and the full boll stages in 2013.

Cultivar	Treatment	Photosynthesis rate (*μ*mol m^−2^ s^−1^) at the full flower stage			Photosynthesis rate (*μ*mol m^−2^ s^−1^) at the full boll stage		
		Leaves	Bracts	Capsule walls	Leaves	Bracts	Capsule walls
Xinluzao 45	CK	28.5 (54.2%)	5.3 (10.1%)	18.8 (35.7%)	16.4 (38.4%)	3.3 (7.8%)	23.1 (53.8%)
	T1	27.3 (52.4%)	5.9 (11.2%)	19.0 (36.4%)	17.7 (42.8%)	3.0 (7.3%)	20.7 (49.9%)
	T2	24.8 (57.8%)	4.4 (10.3%)	13.7 (31.9%)	12.3 (38.3%)	2.4 (7.6%)	17.4 (54.1%)

Xinluzao 33	CK	28.0 (57.3%)	4.3 (8.7%)	16.6 (34.0%)	16.1 (41.5%)	1.9 (4.8%)	20.9 (53.7%)
	T1	30.8 (55.9%)	6.0 (10.9%)	18.3 (33.2%)	12.6 (34.6%)	2.2 (6.0%)	21.7 (59.4%)
	T2	25.3 (57.7%)	3.0 (6.8%)	15.5 (35.5%)	9.1 (37.0%)	1.4 (5.7%)	14.1 (57.3%)

Note: The photosynthetic rates of the three organs were added together to obtain the total photosynthetic rate at each growth stage. The percent contribution of each organ to the total photosynthesis rate is shown within the parentheses.

CK: conventional irrigation; T1: slight deficit irrigation; T2: moderate deficit irrigation.

**Table 3:** Relative contributions of leaf and nonleaf organs to the total surface area of cotton plants grown under different irrigation treatments in 2012 and 2013.

Cultivar	Treatment	2012		2013	
		Leaves (% of total)	Non-leaf organs (% of total)	Leaves (% of total)	Non-leaf organs (% of total)
Xinluzao 45	CK	61.0 ± 0.7^a^	39.0 ± 0.8^c^	61.8 ± 1.3^a^	38.2 ± 0.7^c^
	T1	59.5 ± 1.1^b^	40.5 ± 0.8^b^	58.6 ± 1.1^b^	41.4 ± 1.0^b^
	T2	55.7 ± 0.6^c^	44.3 ± 1.0^a^	56.6 ± 1.2^c^	43.4 ± 1.1^a^

Xinluzao 33	CK	62.6 ± 1.2^a^	37.4 ± 0.7^c^	59.8 ± 1.5^a^	40.2 ± 1.0^b^
	T1	60.4 ± 1.1^b^	39.6 ± 1.1^b^	54.4 ± 1.7^b^	45.6 ± 0.9^a^
	T2	56.9 ± 0.8^c^	43.1 ± 1.3^a^	53.1 ± 1.3^b^	46.9 ± 0.9^a^

Note: Values represent the average surface area between the full flower and the full boll stages. Comparing water treatments within a cultivar, values within a column followed by different letters are significantly different at the 0.05 probability level. Values are means ± SD of three replicates. Abbreviations: CK: conventional irrigation; T1: slight deficit irrigation; T2: moderate deficit irrigation.

**Table 4:** Contribution of nonleaf organs to seed weight in cotton grown under different irrigation treatment in 2012 and 2013.

Variety	Treatment	Seed weight per boll (g)			Contribution (%)		
		Control	Capsule walls plus bracts darkened	Stalk darkened	Capsule walls plus bracts	Stalk	Total contribution
2012							
Xinluzao 33	CK	4.7 ± 0.8^a^	4.0 ± 0.3^a^	4.3 ± 0.4^a^	14.2 ± 1.0^b^	9.2 ± 1.2^b^	23.4 ± 2.7^c^
	T1	4.5 ± 0.4^a^	3.9 ± 0.7^a^	3.9 ± 0.9^a^	14.8 ± 1.1^b^	13.0 ± 2.9^a^	27.8 ± 2.1^b^
	T2	4.4 ± 0.7^a^	3.6 ± 0.3^a^	3.7 ± 0.5^a^	16.8 ± 1.4^a^	14.3 ± 1.6^a^	31.1 ± 3.3^a^
Xinluzao 45	CK	5.0 ± 0.3^a^	4.3 ± 0.9^a^	4.5 ± 0.1^a^	13.4 ± 1.3^b^	9.6 ± 0.5^b^	23.0 ± 1.8^b^
	T1	4.8 ± 0.5^a^	3.5 ± 0.6^b^	4.0 ± 0.2^b^	26.5 ± 2.1^a^	16.7 ± 1.8^a^	43.2 ± 3.9^a^
	T2	4.7 ± 0.4^a^	3.5 ± 0.2^b^	3.9 ± 0.2^b^	24.7 ± 1.9^a^	17.5 ± 2.3^a^	42.2 ± 2.4^a^
Xinluzao 46	CK	5.4 ± 0.2^a^	4.9 ± 0.2^a^	5.1 ± 0.2^a^	10.1 ± 1.7^b^	5.3 ± 1.3^b^	15.4 ± 1.7^b^
	T1	4.3 ± 0.2^b^	3.9 ± 0.9^b^	4.2 ± 0.5^b^	12.7 ± 1.2^a^	10.0 ± 1.1^a^	22.7 ± 2.2^a^
	T2	4.1 ± 0.4^b^	3.5 ± 0.3^b^	3.7 ± 0.6^b^	14.5 ± 2.1^a^	10.3 ± 1.3^a^	24.8 ± 2.6^a^

2013							
Xinluzao 33	CK	5.0 ± 0.4^a^	4.4 ± 0.6^a^	4.7 ± 0.8^a^	18.9 ± 1.1^b^	6.0 ± 0.5^b^	18.6 ± 1.2^b^
	T1	5.5 ± 0.8^a^	4.1 ± 0.3^a^	4.9 ± 0.4^a^	26.0 ± 1.8^a^	10.7 ± 2.3^a^	36.7 ± 2.7^a^
	T2	5.0 ± 0.3^a^	3.6 ± 0.3^b^	4.6 ± 0.2^b^	27.4 ± 1.6^a^	7.6 ± 1.5^b^	35.0 ± 2.2^a^
Xinluzao 45	CK	5.6 ± 0.5^a^	3.9 ± 0.2^a^	5.0 ± 0.6^a^	29.7 ± 0.9^c^	9.9 ± 0.8^b^	39.6 ± 1.1^c^
	T1	5.7 ± 0.7^a^	3.6 ± 0.6^ab^	4.9 ± 1.0^a^	37.1 ± 2.0^a^	15.4 ± 1.2^a^	42.5 ± 2.6^b^
	T2	5.0 ± 0.5^a^	3.3 ± 0.5^bc^	4.3 ± 0.8^a^	34.9 ± 1.2^b^	14.5 ± 1.1^a^	49.4 ± 1.9^a^

Note: Values are means ± SD. Comparing water treatments within a cultivar, values within a column followed by different letters are significantly different at the 0.05 probability level. CK: conventional irrigation; T1: slight deficit irrigation; T2: moderate deficit irrigation.
